# Optical Coherence Tomography Angiography for Diagnosis of Choroidal Neovascularization in Chronic Central Serous Chorioretinopathy after Photodynamic Therapy

**DOI:** 10.1038/s41598-019-45080-8

**Published:** 2019-06-21

**Authors:** Jian-Sheng Wu, San-Ni Chen

**Affiliations:** 1 Department of Ophthalmology, Changhua Christian Hospital, Changhua, Taiwan; 2 Department of Optometry, Chung Shan Medical University, Taichung, Taiwan; 3 Department of Optometry, Da-Yeh University, Changhua, Taiwan; 4 School of Medicine, Chung Shan Medical University, Taichung, Taiwan

**Keywords:** Retinal diseases, Spectrophotometry

## Abstract

The authors evaluated the proportion of choroidal neovascularization (CNV) detected by spectral-domain optical coherence tomography angiography (OCTA) in eyes with chronic central serous chorioretinopathy (CSC) (more than 3 months) with previous treatment via half-dose photodynamic therapy (PDT). All patients were followed up with at least twelve months. Macular angiograms were obtained using spectral-domain OCT (SD-OCT, RTVue XR; Optovue). CNV was defined as flow in the outer retinal slab between the outer plexiform layer and Bruch’s membrane. Clinical characteristics were compared between CNV and non-CNV groups. Seventy eyes of 61 patients (51 male and 10 female) were included. The average age was 46.2 years old. The average duration of symptom was 32.9 months. All patients were treated with half-dose PDT initially. Eleven eyes (15.7%) received more than one session of PDT. CNV was diagnosed in 32 of 70 eyes (45.7%) based on OCTA. Only 6 of the 32 eyes (18.8%) needed intravitreal anti- vascular endothelial growth factor (VEGF) therapy for the exudative activity of CNV. Older age (p = 0.059), larger PDT spot size (p = 0.024), and thinner subfoveal choroidal thickness (p = 0.008) were noted in CNV group. The authors conclude that OCTA reveals high rates of CNV associated with chronic CSC after PDT. Patients in the CNV group had older age, larger PDT spot size, and thinner subfoveal choroidal thickness. OCTA may be considered as a first step in identifying CNV in chronic CSC following PDT.

## Introduction

Chronic central serous chorioretinopathy (CSC) is characterized by widespread retinal pigment epithelium (RPE) atrophic tracks associated with serous retinal detachment^1^. Different from CSC of acute episodes, chronic CSC is related to progressive visual impairment and has been estimated as the fourth most frequent non-surgical retinopathy^2^. In recent years, photodynamic therapy (PDT) has been reported as an effective treatment option for chronic CSC. However, PDT is known to cause secondary RPE changes, choroidal ischemia, choroidal infarction, choriocapillaris hypoperfusion, and choroidal neovascularization (CNV) formation secondary to the hypoxic damage induced by choriocapillaris occlusion at the PDT site^3,4^. Development of CNV is an uncommon complication of chronic CSC following PDT, with the incidence ranging from 2.4% to 16.7% in a meta-analysis study^5^. It is a major cause of reduced visual acuity in chronic CSC^6^ and may require immediate anti-vascular endothelial growth factor (VEGF) therapy^7^. It is challenging to diagnose CNV in chronic CSC because CSC itself can be associated with subretinal fluid (SRF), retinal pigment epithelial detachment (PED), and ill-defined patterns of hyperfluorescence on fluorescein angiography (FA)^8^.

In recent years, optical coherence tomography angiography (OCTA) has been introduced and applied in distinct depth-resolved 3-dimensional visualizations of the choriocapillaris and retinal microvasculature^9^. CNV has been detectable noninvasively based on OCTA. Furthermore, OCTA may provide a method for identifying and guiding treatment of CNV^10^. Previous studies^11,12^ demonstrate that OCTA may be superior to dye-based angiography in the diagnosis of CNV in chronic CSC. The purpose of this study is to evaluate the detection rates of CNV related to chronic CSC after PDT based on OCTA and the associated clinical features.

## Methods

### Patient recruitment

This study followed the Declaration of Helsinki and was approved by institutional review board of Changhua Christian Hospital in Taiwan. Informed consent forms were obtained from all patients to agree receiving OCTA and participating the study. We enrolled patients, most of whom were of Chinese ethnicity, with clinical diagnose of chronic CSC and who had undergone half-dose PDT at Changhua Christian Hospital between April 2007 and September 2016. Half the normal dose of verteporfin (3 mg/m^2^) was infused over 10 minutes, followed by 689-nm laser delivery of 83 seconds at 15 minutes from the commencement of infusion to target the area of choroidal dilation and hyperpermeability in indocyanine green angiography (ICGA). Chronic CSC was defined as at least 3 months of symptoms, including metamorphopsia and blurred vision, with documented CSC clinical features, including RPE changes and SRF located at the macula in spectral-domain optical coherence tomography (SD-OCT) imaging and dye angiography. Clinical examination of both eyes and standardized imaging assessment were used to diagnose chronic CSC. Exclusion criteria were previous intraocular surgery, confluent drusen, pseudodrusen, pathologic myopia (defined as a spherical equivalent refractive error more than −6 diopters), diabetic retinopathy, vitreoretinal diseases, hereditary retinal dystrophies, and prior or active intraocular inflammation.

### CNV defined by OCTA

All participants gave informed consent for OCTA imaging. Macular angiograms (3 × 3 mm) were obtained using SD-OCT (RTVue XR; Optovue). The angiograms were segmented into 4 vascular slabs: superficial retina, deep retina, outer retina, and choriocapillaris. The OCTA software was used to manually adjust the automated segmentation and its depth in the retina and choroid as mentioned previously^11^. Briefly, two automated segmentation lines referencing the outer retina in co-registered OCT B-scans were manually altered to be located at the outer border of the outer plexiform layer (inner boundary) and the level of the Bruch’s membrane (outer boundary). CNV was defined as flow in the outer retinal slab between the outer plexiform layer and Bruch’s membrane. Mean subfoveal choroidal thicknesses were measured in cross-sectional B-scans using standardized SD-OCT.

### Clinical parameters record

Clinical characteristics were recorded and compared between CNV and non-CNV groups, including gender, age at the initial visit of hospital, duration of symptoms, spherical equivalent refractive error, other treatment modalities (intravitreal anti-VEGF injection, micropulse laser, and focal photocoagulation), choroidal vascular hyperpermeability (CVH) in late-phase ICGA, PDT sessions, PDT spot size, period between PDT and OCTA, age at OCTA imaging, persistent SRF at OCTA imaging, duration of follow-up, subfoveal choroidal thickness (SFCT) and best-corrected visual acuity (BCVA).

### Statistics

The categorical factors (e.g., gender and treatment modalities) between the groups were analyzed via Fisher’s exact test or the chi-square test as appropriate. The independent t test or Mann–Whitney U test was used to assess evidence of possible associated continuous factors (e.g., age, duration of symptom, and visual acuity) between groups. Risk factors of CNV were analyzed with univariate and multivariate logistic regression analyses. The correlations between SFCT and predictable variables were calculated by multiple linear regression analyses. Decimal visual acuity was converted to the logarithm of the minimal angle of resolution (LogMAR) for statistics. All analyses were conducted using SPSS v.20.0 software (SPSS, Inc, Chicago, IL). A P value < 0.05 was considered significant.

## Results

### Demographics

**Figure 1:**
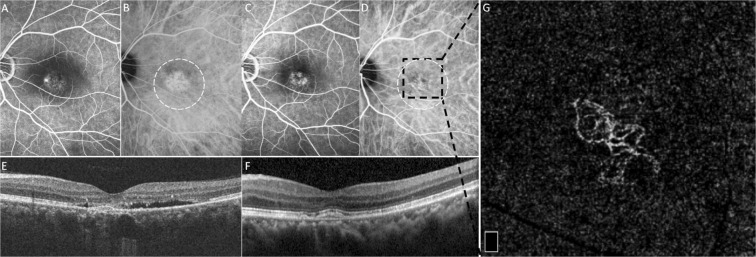
A 55-year-old man had CNV in his left eye based on OCTA, which was located within the previous PDT area. Pre-PDT FA and ICGA (**A**,**B**), and post-PDT FA and ICGA (**C**,**D**) showed hyperfluorescence at macula. Pre-PDT OCT revealed PED and serous RD at macula (**E**). At 3 months after initial occurrence, half-dose PDT with spot size of 3900 μm was applied on the hyperfluorescence area in ICGA (whitish dotted circle in **B** and **D**). Post-PDT OCT disclosed resolution of SRF and persisted PED (**F**). At 44 months after half-dose PDT, a 3 × 3-mm OCTA image at deep retinal slab demonstrated tangled network, which was compatible with CNV and was located within the previous PDT area (**G**).

Seventy eyes of 61 patients (51 male and 10 female) were included. The mean age at the initial visit of hospital was 46.2 years (range, 31 to 66 years). The average duration of symptom was 32.9 months (range, 3 to 183 months). The mean spherical equivalent refractive error was −0.861 diopters (range, −5.750 to 2.625 diopters). CVH in late-phase ICGA was presented in 65 eyes (92.9%). All patients were treated with half-dose PDT initially. Eleven eyes (15.7%, 11/70) received more than one PDT session (range, 1 to 3 times). The mean period from PDT to OCTA was 39.5 months (range, 4 to 138 months). CNV was diagnosed in 32 of 70 eyes (45.7%) based on OCTA. There were 4 eyes (5.7%) with persistent SRF at OCTA imaging. In the CNV group, 6 eyes (18.8%, 6/32) had more than one session of PDT for recurrent SRF, and 6 eyes (18.8%, 6/32) received intravitreal anti-VEGF injection for CNV following PDT. Thirty of the 32 eyes (93.7%) had the CNV based on OCTA located within the previous PDT area (Fig. 1), while only 2 eyes (6.3%) had the CNV based on OCTA outside the initial PDT treatment area.

### Clinical parameters between CNV and non-CNV groups

Comparing the clinical characteristics between CNV and non-CNV groups (Table 1), there were no statistically significant differences in gender, age at the initial visit of hospital, duration of symptoms, spherical equivalent refractive error, other treatment modalities, CVH in late-phase ICGA, PDT sessions, period between PDT and OCTA, persistent SRF at OCTA, nor duration of follow-up. It was marginally significant (p = 0.059) that patients in the CNV group were older when OCTA was performed. Furthermore, larger maximum PDT spot size (p = 0.024) and thinner post-PDT SFCT (p = 0.008) were noted in CNV group. Multivariate logistic regression analyses revealed that the CNV occurrence increased when the maximum PDT spot size was larger than 2400 μm (odds ratio = 3.27; 95% confidence interval [CI], 1.04–10.32; p = 0.043), and when the post-PDT SFCT was thinner than 310 μm (odds ratio = 2.97; 95% CI, 1.02–8.62; p = 0.046) after adjusting for age at OCTA and PDT sessions (Table 2). In addition, age at OCTA and spherical equivalent refractive error were significantly related to the SFCT (R^2^ = 0.276, p < 0.001). It showed that the SFCT decreased 3.24 μm for each year of age at OCTA and increased 23.32 μm for each diopter of spherical equivalent refractive error (Table 3).

**Table 1:** The clinical characteristics of included patients. *P value by Chi-Square test, Fisher’s exact test, one-way ANOVA or Independent *t* test when appropriated. Continuous data are presented as means ± standard deviations. CNV = choroidal neovascularization based on OCTA; VEGF = vascular endothelial growth factor; PDT = photodynamic therapy; CVH = choroidal vascular hyperpermeability; ICGA = indocyanine green angiography; SRF = subretinal fluid; OCTA = optical coherence tomography angiography; SFCT = subfoveal choroidal thickness.

	All eyes (n = 70)	CNV group (n = 32)	non-CNV group (n = 38)	*P value
Gender (Male/Female)	59/11	27/5	32/6	0.985
Age at the initial visit (years)	46.2 ± 7.6	47.8 ± 8.1	44.8 ± 7.2	0.097
Duration of symptoms (months)	32.9 ± 43.5	25.6 ± 38.7	39.2 ± 46.8	0.194
Spherical equivalent (diopters)	−0.861 ± 1.793	−0.816 ± 1.868	−0.898 ± 1.752	0.851
Other treatment modalities				
Anti-VEGF (No/Pre-PDT/Post-PDT)	44/15/11	18/8/6	26/7/5	0.575
Micropulse laser (No/Pre-PDT/Post-PDT)	56/12/2	26/4/2	30/8/0	0.209
Focal photocoagulation (No/Pre-PDT/Post-PDT)	57/11/2	26/6/0	31/5/2	0.362
CVH in late-phase ICGA	65 (92.9%)	31 (96.9%)	34 (89.5%)	0.363
Period of PDT (months)	39.5 ± 34.2	41.6 ± 29.8	37.9 ± 37.9	0.656
Age at OCTA (years)	50.8 ± 7.5	52.6 ± 7.2	49.2 ± 7.6	0.059
Persistent SRF at OCTA (%)	4 (5.7%)	2 (6.3%)	2 (5.3%)	0.860
Duration of follow-up (months)	59.1 ± 29.6	63.0 ± 26.5	55.8 ± 32.0	0.314
PDT session (times)	1.2 ± 0.5	1.3 ± 0.6	1.2 ± 0.4	0.466
Maximum PDT spot size (μm)	2675.7 ± 841.1	2921.9 ± 860.5	2468.4 ± 776.0	0.024
SFCT (μm)	310.7 ± 77.7	284.3 ± 71.5	332.9 ± 76.6	0.008

**Table 2:** Univariable and multivariable analysis of CNV formation in relation to clinical parameters. ^*^P value by logistic regression analysis. CNV = choroidal neovascularization based on OCTA; PDT = photodynamic therapy; OCTA = optical coherence tomography angiography; SFCT = subfoveal choroidal thickness.

	Odds ratio (95% confidence interval) of CNV formation			
	Univariable analysis	*P value	Multivariable analysis	*P value
Older age at OCTA (>50 y/o)	2.56 (0.97–6.72)	0.057	2.50 (0.88–7.13)	0.086
More PDT sessions (>1 time)	1.52 (0.42–5.55)	0.524	1.01 (0.23–4.37)	0.994
Larger PDT spot size (>2400 μm)	3.51 (1.29–9.59)	0.014	3.27 (1.04–10.32)	0.043
Thinner SFCT (<310 μm)	2.79 (1.05–7.41)	0.040	2.97 (1.02–8.62)	0.046

**Table 3:** Multiple linear regression analysis with stepwise approach for SFCT with regard to gender, age at OCTA, spherical equivalent refractive error, PDT sessions and maximum PDT spot size. Age at OCTA and spherical equivalent refractive error were significantly related to SFCT (y = 495.17 − 3.24×_1_ + 23.32×_2_, R^2^ = 0.276, p < 0.001). ^*^P value by multiple linear regression analysis. SFCT = subfoveal choroidal thickness; OCTA = optical coherence tomography angiography.

	Non-standardized coefficient (standard error)	Standard coefficient	t	*P value
Constant	495.17 (59.68)	—	8.298	<0.001
Age at OCTA (year)	−3.24 (1.14)	−0.31	−2.852	0.006
Spherical equivalent (diopter)	23.32 (4.77)	0.54	4.885	<0.001

### BCVA between CNV and non-CNV groups

The LogMAR BCVA improved significantly at 3 months after PDT in both CNV and non-CNV groups, and was maintained as long as 1 year after PDT (Table 4). There were no differences in LogMAR BCVA between CNV and non-CNV groups at any time point.

**Table 4:** Comparisons of sequential logMAR BCVA between groups after PDT. ^*^P value by paired t-test. ^‡^*P* value by Independent t test or Mann-Whitney U test when appropriated. Values are presented as means ± standard deviations. CNV = choroidal neovascularization based on OCTA; BCVA = best-corrected visual acuity; logMAR = logarithm of the minimum angle of resolution; Pre-PDT = BCVA before PDT; Post-PDT = BCVA after PDT.

	Pre-PDT	Post-PDT			
		3 months	6 months	1 year	Final visit
CNV group (n = 32)	0.42 ± 0.38	0.27 ± 0.37	0.26 ± 0.39	0.28 ± 0.42	0.30 ± 0.44
*P value (compared with preceding visit)		0.001	0.943	0.964	0.403
non-CNV group (n = 38)	0.36 ± 0.41	0.24 ± 0.33	0.23 ± 0.37	0.21 ± 0.36	0.21 ± 0.38
*P value (compared with preceding visit)		0.005	0.155	0.303	0.842
^‡^ *P value (CNV vs. non-CNV)*	0.581	0.684	0.763	0.430	0.356

## Discussion

In recent years, CSC has been considered to be one of the spectrum of pachychoroid diseases with features including focal or diffuse increase in choroidal thickness, dilated large choroidal vessels, and choroidal vascular hyperpermeability. Pachychoroid neovasculopathy (PNV), which was initially described by Fung *et al*., refers to the formation of type 1 CNV (sub-RPE) subsequent to CSC^13^. The hypothesized mechanism of CNV occurrence in chronic CSC is that a break appears in Bruch’s membrane due to chronic RPE changes, and long-standing serous PED allows the ingrowth of sub-RPE endothelial cells^14^.

Before the era of OCTA, CNV used to be reported as a rare event secondary to chronic CSC^6,8^ and in chronic CSC after PDT^3,15^. Since the introduction of OCTA, higher incidences of CNV in CSC have been reported. There are several advantages of OCTA over FA in detecting CNV from chronic CSC, including less interference from RPE window defect, and staining from choroidal congestion. Shiragami *et al*. reported 15.6% neovascular CSC in acute CSC and 21.8% in chronic CSC eyes based on OCTA^16^. Bousquet *et al*. reported 35.6% incidence of CNV in chronic CSC eyes with shallow irregular PED^17^. In their study, 8 of 10 eyes with shallow irregular PED and previous PDT treatment had CNV, while only 13 of 49 shallow irregular PED eyes without previous PDT had CNV formation. Quaranta-El M *et al*. reported that OCTA in 7 of 12 eyes with chronic CSC (58%) revealed the presence of CNV corresponding to the ICGA hyperpermeability^18^. In our series of chronic CSC with previous PDT, we also noted a high incidence of CNV formation (45.7%) detected by OCTA.

CNV is a known complication of PDT for chronic CSC. Secondary CNV after PDT in chronic CSC is mainly reported in standard PDT^5^. It has been postulated that a decrease in choroidal perfusion after PDT may increase the risk of CNV development by promoting release of VEGF^19^. Because of the complications reported in CSC patients following standard PDT treatment, a modified half-dose^20^ or half-fluence^21^ protocol, which has been considered safer, was later used to treat chronic CSC. In this study, though no difference in the sessions of half-dose PDT was noted in eyes with CNV, we did notice that the maximum PDT spot size was larger in the CNV group. This indicated that a larger area of choriocapillary hypoperfusion after PDT, causing higher levels of VEGF surge, may induce CNV formation. On the other hand, a more diffuse RPE decompensation and choroidal hyperpermeability may be present in eyes with larger PDT spot size, this rather than PDT, may predispose patients to CNV formation. CNV has also been reported to be a rare complication of laser photocoagulation for CSC (incidence ranging from 1% to 2.8%)^22,23^. There were 13 eyes in our patients had been treated with laser photocoagulation and no difference of laser treatment was found between eyes with or without CNV formation (p = 0.362, Table 1). Otherwise, type 1 CNV has been reported to occur frequently in patient with choroidal vascular hyperpermeability (CVH)^24^. More than 90% eyes with CSC may present CVH in late-phase ICGA^25,26^. We also noted the high prevalence of CVH in our study (65 eyes, 92.9%), but no significant correlation was found between CVH and CNV occurrence (p = 0.363, Table 1).

Our study also found that age at performing OCTA was marginally significantly higher in the CNV group. Older age was a known risk factor for the development of CNV in CSC^8^. It may be because of the aging of the RPE-Bruch’s membrane complex. Another possible explanation is that older patients may have a longer history of chronic CSC, leading to chronic alterations of Bruch’s membrane and RPE, and vulnerability to CNV formation^12^. However, longer duration of symptoms was not found to be a risk factor of CNV in our study.

Choroidal thickness after PDT was found to be statistically thinner in the CNV group, though still thicker than the average thickness in the normal population^27^. The SFCT depends on various physiological factors and varies with age, gender, refraction, or axial length^27–29^. In addition, a reduction in choroidal thickness of 16–21% at 3 months has been reported following half-dose PDT for chronic CSC^30,31^. Thus, the relationship between SFCT and predictable variables, including gender, age at OCTA, spherical equivalent refractive error, PDT sessions and maximum PDT spot size, was analyzed by multiple linear regression (Supplementary Table 1). Only age at OCTA and spherical equivalent refractive error were significantly related to the SFCT (R^2^ = 0.276, p < 0.001, Table 3). Furthermore, we used multivariate logistic regression to elucidate the correlation between SFCT and the occurrence of CNV, and SFCT was still significantly negatively related to the occurrence of CNV (odds ratio = 0.88 for every 10 μm increase of SFCT, 95% CI, 0.81–0.97; p = 0.007) after adjustment for age at OCTA, gender, PDT sessions, PDT spot size and spherical equivalent refractive error (Supplementary Table 2). As CNV was a complication more often noted in patients who are older^6^, when the choroid is supposed to become thinner with aging changes^29^, PDT-induced permanent choroidal thickness reduction may similarly increase the chance of CNV formation.

In this study, it was not unexpected to note that most CNV was located within the area of previous PDT. Since PDT was applied at the area of choroidal congestion and RPE leakage, pre-existing defects in Bruch’s membrane due to chronic RPE changes and long-standing serous PED^13^, as well as choriocapillary thinning secondary to the underlying choroidal congestion^32^, may be a preceding factor for the development of CNV. It may be possible that CNV has already existed before PDT. Half-dose PDT, though supposedly safer, may further exacerbate the already compromised choriocapillaries, and increase the incidence of CNV.

Our study demonstrated that there were no differences in either initial or final BCVA between the CNV and non-CNV groups, which was different from a recent study^16^, in which initial and final BCVA were both significantly worse in eyes with CNV. However, their study included both eyes with acute and chronic CSC; in contrast, only eyes with chronic CSC were included in our study. Since most eyes in the CNV group in our study remained silent, and those eyes with exudative activity related to CNV were managed with intravitreal anti-VEGF injection, no significant effect of visual deterioration of CNV was observed.

The main limitation of this study was a possible selection bias because these patients were recruited from clinic follow-up. In addition, owing to lack of pre-PDT OCTA, it is difficult to tell if the CNV detected by OCTA occurred before or after half-dose PDT treatment. Other limitations included heterogeneous treatment histories, small OCTA scan area, and a relatively small number of patients. Further large and prospective studies are needed to explore the relationship of CNV and chronic CSC after half-dose PDT.

In conclusion, OCTA reveals high rates of CNV associated with chronic CSC after half-dose PDT. Patients in the CNV group had older age, larger PDT spot size, and thinner subfoveal choroidal thickness. OCTA may be considered as a first step in identifying CNV in chronic CSC after half-dose PDT.

## Supplementary information

Supplementary Information
