# Supplementary material for: Optical Coherence Tomography Angiography for Diagnosis of Choroidal Neovascularization in Chronic Central Serous Chorioretinopathy after Photodynamic Therapy
Source: Sci Rep. 2019 Jun 21;9:9040. doi: 10.1038/s41598-019-45080-8 (PMC6588615; doi:10.1038/s41598-019-45080-8)
Supplement: Supplementary file 1 — Supplementary Information [file 41598_2019_45080_MOESM1_ESM.pdf]

Optical Coherence Tomography Angiography for Diagnosis of Choroidal Neovascularization in Chronic Central Serous Chorioretinopathy after Photodynamic Therapy

Jian-Sheng Wu, MD,[1,2,3] San-Ni Chen, MD[1,3,4]

1. Department of Ophthalmology, Changhua Christian Hospital, Changhua, Taiwan

2. Department of Optometry, Chung Shan Medical University, Taichung, Taiwan

3. Department of Optometry, Da-Yeh University, Changhua, Taiwan

4. School of Medicine, Chung Shan Medical University, Taichung, Taiwan

Correspondence and requests for materials should be addressed to S.-N.C.

Address: Department of Ophthalmology, Changhua Christian Hospital, No. 135, Nanxiao St. Changhua City, Taiwan, Republic of China.

Telephone No: 886-4-7238595 ext 4454

E-Mail address: 108562@cch.org.tw

Fax No: 886-4-7228289

No authors have a proprietary interest

	Non-standardized coefficient (standard error)	Standard coefficient	t	*P value
**Constant**	453.958 (66.167)	-	6.861	0.887
**Gender (Male)**	24.632 (23.060)	0.116	1.068	0.289
**Age at OCTA (year)**	-3.335 (1.201)	-0.324	-2.778	0.007
**Spherical equivalent (diopter)**	22.335 (4.862)	0.515	4.594	<0.001
**PDT session (time)**	8.873 (18.112)	0.057	0.490	0.626
**Maximum PDT spot size (μm)**	0.005 (0.011)	0.056	0.475	0.636

Supplementary Table 1. Multiple linear regression analysis for SFCT with regard to gender, age at OCTA, spherical equivalent refractive error, PDT sessions and maximum PDT spot size. *P value by multiple linear regression analysis. SFCT = subfoveal choroidal thickness; OCTA = optical coherence tomography angiography; PDT = photodynamic therapy.

	Odds ratio (95% confidence interval) of CNV formation			
	Univariable analysis	*P value	Multivariable analysis	*P value
**Gender (Male)**	1.10 (0.28-3.69)	0.985	1.12 (0.24-5.30)	0.887
**Age at OCTA (/year)**	1.07 (0.99-1.14)	0.064	1.02 (0.94-1.11)	0.573
**Spherical equivalent (/diopter)**	1.03 (0.79-1.34)	0.849	1.23 (0.85-1.79)	0.272
**PDT session (/time)**	1.46 (0.56-3.82)	0.444	0.77 (0.23-2.59)	0.667
**Maximum PDT spot size (/100μm)**	1.07 (1.01-1.14)	0.027	1.09 (1.01-1.18)	0.039
**SFCT (/10μm)**	0.92 (0.85-0.98)	0.014	0.88 (0.81-0.97)	0.007

Supplementary Table 2. SFCT was significantly negatively related to the occurrence of CNV (odds ratio=0.88 for every 10μm increase of SFCT, 95% CI, 0.81-0.97; p=0.007) after adjustment for gender, age at OCTA, spherical equivalent refractive error, PDT sessions and PDT spot size. *P value by logistic regression analysis. CNV = choroidal neovascularization based on OCTA; PDT = photodynamic therapy; OCTA = optical coherence tomography angiography; SFCT = subfoveal choroidal thickness.
